# Gray and White matter microstructural alterations in major depressive disorder: a multi-center diffusion imaging study

**DOI:** 10.1038/s41398-026-03916-8

**Published:** 2026-02-19

**Authors:** Kento Takahashi, Taro Suwa, Yujiro Yoshihara, Yusuke Kyuragi, Naoya Oishi, Harumasa Takano, Takamasa Noda, Jinichi Hirano, Momoko Hatakoshi, Yuzuki Ishikawa, Jun Miyata, Hiroyuki Igarashi, Hiroyuki Kanno, Shingo Murakami, Masaru Mimura, Kazuyuki Nakagome, Toshiya Murai

**Affiliations:** 1 Department of Psychiatry, Graduate School of Medicine, Kyoto University, Kyoto, 606-8507 Japan; 2 Human Brain Research Center, Graduate School of Medicine, Kyoto University, Kyoto, 606-8507 Japan; 3 Integrative Brain Imaging Center, National Center of Neurology and Psychiatry, Tokyo, 187-8551 Japan; 4 Department of Psychiatry, National Center of Neurology and Psychiatry, Tokyo, 187-8551 Japan; 5 Department of Neuropsychiatry, Keio University School of Medicine, Tokyo, 160-8582 Japan; 6 Department of Psychiatry, Aichi Medical University, Nagakute, Aichi 480-1195 Japan; 7 National Center of Neurology and Psychiatry, Tokyo, 187-8551 Japan

**Keywords:** Depression, Neuroscience

## Abstract

Diffusion imaging techniques have been widely used to investigate alterations in brain microstructure associated with major depressive disorder (MDD). Due to its technical limitations, diffusion tensor imaging (DTI)-based studies have often been restricted to evaluating white matter (WM), and analyses of gray matter (GM) microstructural changes using advanced diffusion models remain insufficient. Additionally, many of these studies concentrate on region-specific associations with symptoms rather than a comprehensive assessment of broader microstructural changes. In this study, we employed neurite orientation dispersion and density imaging (NODDI) and DTI to investigate GM and WM microstructural changes at both whole-brain and regional levels. Data were collected from 159 MDD patients and 112 healthy controls across multiple centers. Our findings revealed significantly increased mean free water fraction (FWF) in GM, elevated mean orientation dispersion index (ODI) in WM, and decreased fractional anisotropy (FA) in WM among MDD patients compared to healthy controls. Furthermore, the mean FA of WM exhibited a negative correlation, and the mean ODI of WM showed a positive correlation with illness duration. No significant correlations were observed between diffusion indices and Hamilton Depression Rating Scale (HAMD-17) scores. Gray matter-based spatial statistics demonstrated increased FWF in several GM regions, including the frontal lobes, temporal lobes, and limbic system. Tract-based spatial statistics revealed widespread reductions in FA across WM in MDD patients. These findings suggest that microstructural tissue disorganization may underlie the pathophysiology of MDD, emphasizing the need for future research to link neuroimaging findings with underlying biological mechanisms.

## Introduction

Major depressive disorder (MDD) is a psychiatric condition characterized by persistent feelings of sadness, decreased motivation, and loss of interest, and it is one of the leading causes of global disease burden [1]. Alterations in gray matter (GM) and white matter (WM) microstructure have been implicated in the pathophysiology of MDD. Postmortem studies have demonstrated reductions in neuronal size and glial cell density within GM [2], as well as decreases in both the number and density of oligodendrocytes [3–5**]**. In WM, reductions in myelin [6, 7**]** and oligodendrocyte cell body size have also been reported [8]. Animal studies utilizing models of MDD suggest dendritic atrophy and spine loss in GM [9], accompanied by decreases in mature oligodendrocytes [10] and astrocytes [11]. Additionally, chronic stress-induced impairments in synaptic plasticity have been shown to be reversible with antidepressant treatment [12]. In WM, demyelination and reduced myelin sheath thickness have been observed in MDD animal models [13, 14], and antidepressant treatments have been reported to prevent WM damage, restoring both myelin integrity and oligodendrocyte numbers [15, 16].

In patients currently experiencing depressive symptoms, in vivo neuroimaging provides a non-invasive method for collecting and analyzing large datasets, offering valuable insights into brain microstructural changes associated with MDD. Diffusion tensor imaging (DTI) is a widely used technique for visualizing the diffusion properties of water molecules within tissues, particularly in studying WM structures [17**–**19]. Fractional anisotropy (FA), the most commonly used DTI index, quantifies the directional restriction of water diffusion, where higher FA values indicate increased WM directionality. Multi-center studies and meta-analyses have consistently reported FA reductions in several brain regions, such as the corpus callosum and corona radiata, among patients with MDD [20**–**24].

DTI employs a single-component model that approximates the diffusion within each voxel as an ellipsoid and assumes that water molecule diffusion follows a Gaussian distribution. Consequently, DTI is limited in its ability to capture multifaceted structural changes, particularly in GM, which has a more complex fiber architecture than WM [25**–**27]. Neurite orientation dispersion and density imaging (NODDI) addresses this limitation by modeling the water diffusion properties of both GM and WM through three distinct compartments: intra-neurite (axons and dendrites), extra-neurite (the space defined by somas and glial cell membranes), and free water (cerebrospinal fluid and edema) [28]. Combining these multiple modalities, each with unique characteristics, can provide a more comprehensive understanding of GM and WM pathophysiology at a histological level compared to single-modality studies. NODDI calculates several key indices, including the neurite density index (NDI), which reflects the fraction of intra-neurite volume; the orientation dispersion index (ODI), which characterizes the angular variation of neurites; and the free water fraction (FWF), representing the proportion of free water present in the tissue (Supplementary Fig. S1). To our knowledge, only two studies have utilized NODDI in MDD research to date [29, 30]. One study reported reduced NDI in regions such as the right superior temporal cortex and bilateral insular cortex, reduced ODI in the left thalamus and left occipital cortex, and increased ODI in regions such as the bilateral superior longitudinal fasciculus and left posterior thalamic radiation [29]. Another study investigated the effects of ketamine on WM in treatment-resistant MDD and found a significant decrease in NDI within occipitotemporal WM pathways following serial ketamine infusion; this correlated with improvements in anhedonia, with no significant changes observed in ODI or FA [30]. These findings suggest that MDD is associated with changes in neurite density and orientation, though the results have been inconsistent and limited to smaller cohorts.

Previous neuroimaging studies on MDD have predominantly focused on the association between MDD diagnosis or symptoms and specific brain regions. However, limited discussion exists regarding the nature of microstructural changes on a whole-brain level. The involvement of the endocrine and immune systems in the pathophysiology of MDD is well-established [31**–**35], with increasing evidence suggesting that excessive cortisol levels and neuroinflammation may contribute to microstructural alterations in MDD [36**–**39]. Given the systemic nature of hormonal and inflammatory mediators, it is plausible that microstructural changes in MDD occur not only at region-specific or circuit-specific levels but also across the whole brain. Indeed, some studies have reported decreased mean FA values across the entire WM in MDD [20, 40, 41].

In this study, we hypothesized that microstructural changes occur across the whole brain in patients with MDD. To test this hypothesis, we examined microstructural alterations in both GM and WM using DTI and NODDI, utilizing a multi-center MRI dataset comprising patients with MDD and healthy controls (HC). We investigated global differences in diffusion indices between patients with MDD and HC. For indices showing significant differences, we further explored their associations with clinical parameters.

## Methods

### Participants

Participants were recruited from the Longitudinal MRI study Identifying the Neural Substrates of Remission/Recovery in Mood Disorders (L/R Study), an observational study that is part of the Brain MINDS/Beyond project [42]. The present study included 76 patients with MDD and 55 HC from Kyoto University Hospital (KUHP), 83 patients with MDD, and 57 HC from the National Center of Neurology and Psychiatry (NCNP), for a total of 159 patients with MDD and 112 HC. The inclusion criteria were a diagnosis of MDD according to the structured interview Mini International Neuropsychiatric Interview (MINI) [43], age between 20 and 85 years, and a Hamilton Depression Rating Scale (HAMD-17) [44] score of 8 or higher at the time of participation. Key exclusion criteria included a history of substance use disorder or alcohol abuse, suicidal ideation or attempts, dementia, MRI contraindications, and serious mental or physical conditions.

For the clinical data of the MDD group, illness severity was assessed using HAMD-17 scores, and illness duration was evaluated based on the interval between age at onset and age at the time of imaging acquisition.

The study was approved by the Medical Ethics Committees of Kyoto University and the National Center Hospital, National Center of Neurology and Psychiatry. All participants provided written informed consent, and the study adhered to the principles outlined in the Declaration of Helsinki.

### MRI acquisition

MRI data were acquired using 3 T MRI scanners at both KUHP and NCNP. At KUHP, a MAGNETOM Verio (Siemens, Erlangen, Germany) scanner was used, while at NCNP, a MAGNETOM Skyra fit (Siemens, Erlangen, Germany) scanner was utilized. Imaging protocols adhered to the Harmonized Protocol for Neuroimaging in Psychiatry (HARP) [45]. T1-weighted images were obtained using a 3-dimensional magnetization-prepared rapid gradient echo (3D-MPRAGE) sequence with the following parameters: repetition time (TR), 2500 ms; echo time (TE), 2.18 ms; inversion time (TI), 1000 ms; flip angle, 8°; field of view (FOV), 256 × 240 mm; matrix size, 320 × 300; spatial resolution, 0.8 × 0.8 × 0.8 mm; and slice per slab, 224. T2-weighted images (T2WIs) were acquired with a 3D T2 Sampling Perfection with Application-optimized Contrasts using different flip angle Evolutions (3D T2-SPACE) sequences. The parameters were as follows: TR, 3200 ms; TE, 565 ms for Verio and 564 ms for Skyra fit; all other parameters were identical to those of the 3D-MPRAGE. Diffusion-weighted images were obtained using a spin-echo echo-planar imaging (EPI) sequence with the following parameters: TR, 3600 ms; TE, 89.0 ms; flip angle, 90°/180°; FOV, 204 × 204 mm; matrix size, 120 × 120; spatial resolution, 1.7 × 1.7 × 1.7 mm; slice number, 84; and b-values of 0/700/2000 s/mm². Diffusion MRI data were collected with reverse phase-encoding pairs (anterior-posterior [AP] and posterior-anterior [PA]), consisting of 67 diffusion directions for AP and 68 for PA.

### MRI data preprocessing

All MRI data were preprocessed using the Human Connectome Project (HCP) pipeline [46]. Briefly, T1-weighted images underwent gradient distortion correction [47], followed by registration to Montreal Neurological Institute (MNI) space using FLIRT [48**–**50] and FNIRT [51] within the FMRIB Software Library (FSL) version 6.0.5 (FSL; Oxford Centre for Functional MRI of the Brain, Oxford, UK; www.fmrib.ox.ac.uk/fsl). For diffusion-weighted images, series-wise intensity normalization of the b0 images was performed to ensure consistent signal magnitude across opposing phase-encoding acquisitions (details in Supplementary Materials). This was followed by correction of EPI distortions using FSL’s “topup” tool [52] and correction of eddy currents and motion artifacts using FSL’s “eddy” tool [53]. Alignment to T1-weighted images was achieved using FLIRT and the boundary-based registration (BBR) tool in FreeSurfer (version 6.0.1) [48**–**50].

### DTI and NODDI

FA maps were generated using FSL’s DTIFIT tool. NODDI models brain microstructure by dividing it into three compartments: the intra-neurite compartment, modeled as restricted diffusion; the extra-neurite compartment, modeled as anisotropic hindered diffusion; and the free water compartment, modeled as isotropic diffusion. The model yields key indices such as NDI, which reflects the intra-neurite volume fraction; ODI, which quantifies the angular variation of the neurites; and FWF, which represents the proportion of unbound water present within the tissue. These indices were derived using accelerated microstructure imaging via convex optimization (AMICO, version 2.0.1) [54], a method that linearly approximates the NODDI model to significantly improve computational speed without compromising accuracy. Brain masks were created using Synthstrip [55] in FreeSurfer (version 7.4.1), a deep learning-based skull-stripping tool that enables rapid and precise brain extraction. All index maps were visually inspected for quality assurance. Examples of the fitted index maps are provided in Supplementary Fig. S2.

### TBSS and GBSS

To evaluate WM diffusion indices, we utilized tract-based spatial statistics (TBSS, version 1.2) in FSL [56]. Briefly, FA images of all participants were registered to a standard FA image (FMRIB58_FA) in Montreal Neurological Institute (MNI) space, creating a mean FA image, which was then used to generate an FA skeleton image. This mean FA skeleton image was thresholded at 0.2 to create a binary mask, onto which the nearest voxel values of FA maps for individual subjects were projected. The same projection vectors were applied to NDI, ODI, and FWF maps for all subjects (Supplementary Fig. S3A).

For GM analysis, we employed gray matter-based spatial statistics (GBSS) [57], an approach analogous to TBSS but adapted for GM. First, GM segmentation was performed on T1-weighted images using FSL’s FAST tool [57–60]. Segmented GM images were registered to MNI space using transformation matrices generated by the HCP pipeline, creating a mean GM skeleton. This skeleton was thresholded at 0.2 to produce a binary mask, onto which NDI, ODI, and FWF index maps were projected using linear and non-linear transformation matrices from the HCP pipeline (Supplementary Fig. S3B).

### Statistical analyses

Statistical analyses, excluding voxelwise analyses, were conducted using R (version 4.3.3). To account for potential site-specific batch effects inherent in multi-site diffusion imaging data, we employed the ComBat method for data harmonization [61]. The ComBat function was implemented using R. The application of ComBat was tailored based on the type of analysis. For global metrics (e.g., mean NDI of GM), the metric values were first extracted from the unharmonized images, and then ComBat was applied to these extracted values to adjust for site effects before performing statistical analysis. For voxelwise analyses (TBSS and GBSS), ComBat was applied directly to the unharmonized index maps to produce harmonized maps, which were subsequently used for statistical comparisons. In analyses involving both HC and MDD groups, group, age, and sex were included in the site-effect estimation to preserve the effects of these variables. In analyses involving only the MDD group, age, sex, HAMD-17 score, and illness duration were included in the site-effect estimation. Due to missing illness duration data for seven patients, 152 patients were included in MDD-only analyses.

For basic demographic comparisons, age differences between groups were tested using the Wilcoxon rank-sum test due to the non-normal distribution determined using the Kolmogorov-Smirnov test. Differences in sex ratios between groups were tested using the chi-square test. Statistical significance was set at p < 0.05.

Following previous studies [40, 41, 62–65], we calculated the mean values of each index for GM and WM skeletons for each subject. For GM indices, group comparisons of mean NDI, ODI, and FWF between the HC and MDD groups were conducted using age and sex as covariates, with statistical significance set at p < 0.0166 (0.05/3 indices). Similarly, for WM indices, group comparisons were performed for mean NDI, ODI, FWF, and FA with statistical significance set at p < 0.0125 (0.05/4 indices).

For each diffusion index showing significant group differences in the aforementioned analyses, we conducted the following follow-up analyses. We investigated whether interactions existed between diagnosis and age or sex. Using linear models adjusted for sex, we analyzed the interaction between diagnosis and age on diffusion indices. Similarly, the diagnosis-by-sex interaction was examined, controlling for age. We also explored the correlation between diffusion indices and HAMD-17 scores, as well as illness duration, using linear models adjusted for age and sex. The statistical threshold was set at p < 0.05.

Additionally, for each diffusion index showing significant group differences in global measures, voxelwise analyses of GBSS and TBSS were performed to identify the specific brain regions contributing to these global differences. We used FSL’s randomize tool [66], a permutation-based nonparametric inference tool, to conduct unpaired t-tests between HC and patients with MDD, adjusting for age and sex, with 10,000 permutations. The statistical threshold was set at p < 0.05, and the family-wise error (FWE) rate was corrected using threshold-free cluster enhancement (TFCE) [67]. Significant regions were identified using the Harvard-Oxford cortical and subcortical structural atlases for GM and the JHU ICBM-DTI-81 White-Matter Labels for WM.

## Results

### Demographics and clinical data

Detailed demographic data are presented in Table 1. No significant differences in age or sex were observed between the HC and MDD groups.

**Table 1:** Demographic and clinical characteristics of the participants.

	HC	MDD	*p* value
Number of subjects	112	159	
Age, mean years (SD)	41.6 (13.7)	43.7 (14.1)	0.251
Gender (male/female)	47/65	76/83	0.409
HAMD-17, mean (SD)	--	15.7 (6.18)	--
Illness duration, mean years (SD)	--	9.09 (8.04)	--

Group differences in age were tested using the Wilcoxon rank sum test, while group differences in sex ratio were tested using the chi-squared test.

*HC* healthy control, *MDD* major depressive disorder, *SD* standard deviation, *HAMD-17* hamilton depression rating scale 17 items.

### Group differences in diffusion indices

We observed a significant increase in the mean FWF of GM in the MDD group compared to the HC group (Table 2). Additionally, the MDD group showed a significant increase in the mean ODI and a significant decrease in the mean FA of WM compared to the HC group (Table 3).

**Table 2:** Multiple linear regression analysis comparing gray matter (GM) diffusion indices between groups, adjusted for age and sex.

Parameters	HC, mean (SD)	MDD, mean (SD)	β (SE)	*t* value	*p* value
NDI	0.5138 (0.0179)	0.5151 (0.0179)	−0.0314 (0.0016)	−0.726	0.468
ODI	0.5039 (0.0081)	0.5064 (0.0084)	0.0961 (0.0009)	1.856	0.0646
FWF	0.1373 (0.0326)	0.1501 (0.0380)	0.1125 (0.0009)	2.649	0.0086*

*HC* healthy control, *MDD* major depressive disorder, *SD* standard deviation; β, standardized regression coefficient for group variable, adjusted for age and gender, *SE* standard error.

*p < 0.0167.

**Table 3:** Multiple linear regression analysis comparing white matter (WM) diffusion indices between groups, adjusted for age and sex.

Parameters	HC, mean (SD)	MDD, mean (SD)	β (SE)	*t* value	*p* value
NDI	0.6277 (0.0222)	0.6223 (0.0230)	−0.1369 (0.0027)	−2.368	0.0186
ODI	0.2275 (0.0086)	0.2307 (0.0091)	0.1149 (0.0008)	2.612	0.0095*
FWF	0.0862 (0.0086)	0.0868 (0.0092)	−0.0051 (0.0010)	−0.093	0.926
FA	0.4736 (0.0120)	0.4680 (0.0145)	−0.1730 (0.0015)	−3.218	0.0015*

*HC* healthy control, *MDD* major depressive disorder, *SD* standard deviation; β, standardized regression coefficient for group variable, adjusted for age and gender, *SE* standard error.

*p < 0.01.

Further analyses focused on the mean FA and mean ODI of WM, and the mean FWF of GM, which exhibited significant group differences.

### Interaction between diagnosis and age/sex on diffusion indices

No significant interactions were found between diagnosis and age for the mean FWF of GM (F4, 266 = 1.454, p = 0.229), the mean ODI of WM (F4, 266 = 0.861, p = 0.354), or the mean FA of WM (F4, 266 = 0.967, p = 0.326) (Supplementary Fig. S4A). Similarly, no significant interactions between diagnosis and sex were identified for the mean FWF of GM (F4, 266 = 0.919, p = 0.339), the mean ODI of WM (F4, 266 = 1.631, p = 0.203), or the mean FA of WM (F4, 266 = 3.031, p = 0.082) (Supplementary Fig. S4B).

### Correlation of diffusion indices with symptom severity

No significant correlations were found for the mean FWF of GM (β = −0.028, SE = 0.0591, p = 0.622), the mean ODI of WM (β = −0.035, SE = 0.0637, p = 0.584), or the mean FA of WM (β = −0.021, SE = 0.0757, p = 0.769).

### Correlation of diffusion indices with illness duration

**Fig. 1:**
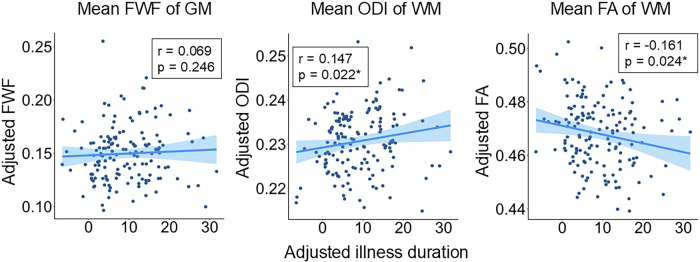
Scatter plots and regression lines depicting the correlation between illness duration and diffusion indices. The X-axis represents the illness duration adjusted for age and sex, and the Y-axis represents the diffusion indices adjusted for age and sex.

We identified a positive correlation between the mean ODI of WM and illness duration (β = 0.139, SE = 0.0684, p = 0.042). Additionally, a negative correlation was found between the mean FA of WM and illness duration (β = −0.182, SE = 0.0807, p = 0.019). No significant correlation was observed between the mean FWF of GM and illness duration (β = 0.037, SE = 0.0642, p = 0.551) (Fig. 1).

### Skeleton-based voxelwise analysis

**Fig. 2:**
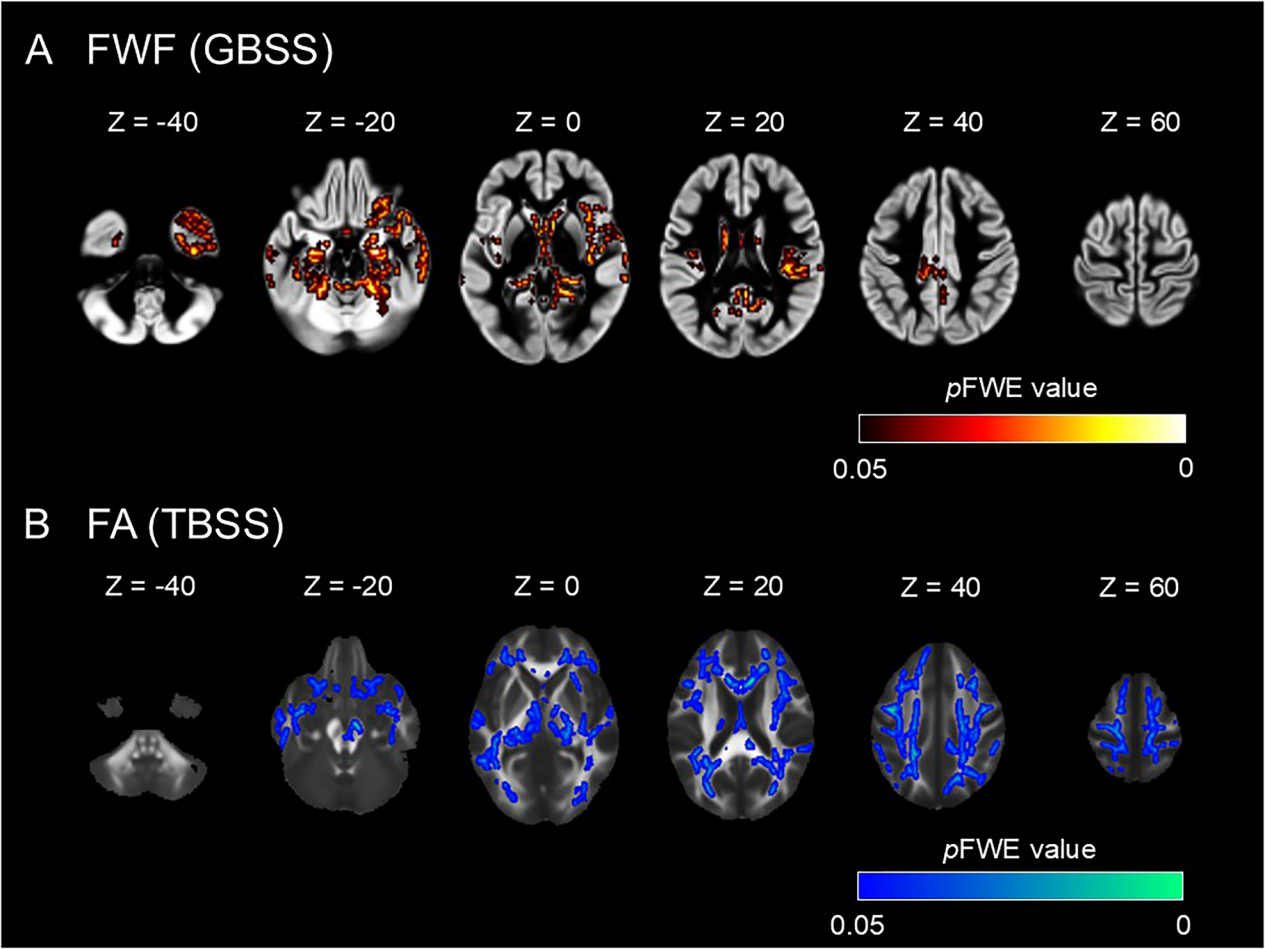
Group comparisons of diffusion indices between the healthy controls and the patients with MDD. **A** GBSS revealed increases in FWF in the MDD group (red-yellow voxels). **B** TBSS revealed decreases in FA in the MDD group (blue-light blue voxels). The ‘tbss_fill’ command in FSL was used to enhance visibility of the significant tracts.

In GBSS analysis, the MDD group exhibited increased FWF values in several regions, including the bilateral frontal lobes, temporal lobes, insular cortex, hippocampus, and amygdala (Fig. 2A, Supplementary Table S1). In TBSS, the MDD group demonstrated FA reductions in widespread WM regions, including uncinate fasciculus, cingulum, anterior limb of internal capsule, and corpus callosum (Fig. 2B, Supplementary Table S2). No voxels with significant differences were identified for ODI values.

### Other DTI indices

To enable a more comprehensive comparison with previous DTI studies and provide a full-tensor characterization of WM microstructure [68, 69], we conducted exploratory analyses on other DTI-derived indices: mean diffusivity (MD), axial diffusivity (AD), and radial diffusivity (RD). At the whole-brain level, the MDD group exhibited a significant increase in mean MD of WM (β = 0.132, SE = 1.69 × 10^−6^, p = 0.0279) and mean RD of WM (β = 0.154, SE = 1.80 × 10^−6^, p = 0.0101) compared to the HC group. No significant difference was found in mean AD of WM (β = 0.0447, SE = 1.90 × 10^−6^, p = 0.417). TBSS analyses of the significant metrics (MD and RD) revealed widespread increases in both MD and RD (pFWE < 0.05) in the MDD group compared to the HC group. These increases were observed in various WM tracts, including the corpus callosum, anterior limb of the internal capsule, uncinate fasciculus, and cingulum (Supplementary Fig. S5, Supplementary Tables S3 and S4).

## Discussion

This study identified an increase in mean FWF values across the entire GM, a decrease in mean FA values, and an increase in mean ODI values across the entire WM in patients with MDD. There were no significant correlations between HAMD-17 scores and the mean FWF of GM, mean ODI, or mean FA of WM. Illness duration was positively correlated with the mean ODI of WM and negatively correlated with the mean FA of WM. Voxelwise analyses revealed widespread reductions in FA values in WM and increases in FWF values in several GM regions, including the frontal and temporal lobes and the insular cortex.

### Gray matter microstructural alteration

This is the first study to reveal an increased proportion of free water in the GM of patients with MDD. The increase in the free-water component may suggest neuroinflammation [70**–**72]. Previous human and animal studies have reported associations between depression and elevated levels of inflammatory cytokines [35, 73]. Moreover, increased proinflammatory signaling has been linked to a higher proportion of free water [72].

Skeleton-based analyses showed increased FWF values in several regions, including the bilateral frontal lobes, temporal lobes, insular cortex, hippocampus, and amygdala. Although no previous studies have specifically investigated FWF in GM among patients with MDD, structural neuroimaging studies have reported reduced cortical thickness in regions such as the orbitofrontal cortex, insula, cingulate cortex, and fusiform gyrus [74], as well as volume reductions in the parahippocampal gyrus [75], frontal lobe, temporal lobe, and limbic system [76] in patients with MDD. Additionally, fMRI studies have shown altered resting-state connectivity in regions such as the hippocampus and insular cortex in patients with depression [77]. These regions overlap with those exhibiting increased FWF values in our study.

Significantly increased FWF was observed across several GM regions, many of which are key components of emotional and cognitive networks implicated in MDD pathophysiology. Specifically, the orbitofrontal cortex has been linked to core MDD symptoms such as anhedonia and negative bias [78]. The temporal pole is implicated in pathological affect regulation [79]. The insular cortex is associated with malfunction of interoception [80]. The amygdala, a crucial node in the network controlling emotion, has been reported to be hyperactive to negative stimuli in MDD [81]. Furthermore, the hippocampus is consistently implicated in the cognitive and memory dysfunction often observed in MDD [82]. Given that an increase in FWF may indicate neuroinflammatory changes, the observed FWF alterations in these specific regions suggest that neuroinflammatory processes within these critical emotion- and cognition-regulating areas, and the resultant functional disturbances, may be closely associated with the clinical presentation of MDD.

Previous research has documented reduced NDI in the right superior temporal cortex and bilateral insular cortex, alongside reduced ODI in the left thalamus and left occipital cortex [29]. These findings were not replicated in our study, which could be attributed to our larger sample size, potentially reducing variability and enhancing the robustness of our results.

### White matter microstructural alteration

Our study identified an increase in mean ODI values and a decrease in mean FA values in the WM of patients with MDD. The reduction in mean FA values aligns with previous reports [20, 40, 41]. Voxelwise analysis revealed widespread reductions in FA across WM tracts, involving several key WM tracts integral to MDD pathophysiology. Specifically, the observed FA reductions encompassed the uncinate fasciculus, cingulum, anterior limb of the internal capsule, and corpus callosum. The uncinate fasciculus connects key regions of the cortico-limbic circuit, and its disruption has consistently been implicated in MDD pathophysiology [83]. The cingulum is considered a core element of the reward pathway, and alterations in this tract are thought to be associated with symptoms like anhedonia [84]. Furthermore, the anterior limb of the internal capsule connects the thalamus to the cingulate gyrus and prefrontal cortex, playing a critical role in motivation, reward, decision-making, and emotion [85]. The corpus callosum is responsible for interhemispheric communication, and its microstructural disruption has been suggested to be associated with the cognitive impairment frequently reported in MDD [86]. While we observed increased mean ODI values in WM, the voxelwise analysis did not identify significant region-specific ODI changes, indicating that ODI alterations may not be localized to specific regions.

Although no prior studies have directly compared mean ODI values between MDD and control groups, voxel-based analyses have reported increased ODI in regions such as the bilateral superior longitudinal fasciculus and left posterior thalamic radiation in patients with MDD [29]. The lack of replication of voxel-level ODI changes in our study could be attributed to the larger sample size, which likely reduced variability.

FA is widely recognized as an indicator of WM connectivity [17] and its reduction is generally considered to reflect structural disruption due to various pathologies. The increase in ODI can be interpreted as a decrease in the coherence of neurite orientation, although the underlying pathology is multifactorial [70]. Accumulating evidence supports the involvement of neuroinflammation in the pathophysiology of depression [34**–**36]. Specifically, animal studies have reported associations between increased ODI and pathologies such as demyelination, microglial activation, astrogliosis, and axonal degeneration [87**–**89]. The observed changes in diffusion indices in this study, namely reduced FA and increased ODI, may reflect demyelination, glial cell infiltration, or axonal injury secondary to neuroinflammatory processes in MDD.

The additional exploratory analyses of MD, AD and RD revealed an increase in mean MD of WM and mean RD of WM in the MDD group compared to the HC group. Furthermore, voxelwise analysis showed that the increases in both MD and RD were widespread, encompassing tracts such as the uncinate fasciculus, the cingulum, the anterior limb of the internal capsule, and the corpus callosum. Increases in MD and RD in the MDD group are consistent with findings reported in previous studies [20, 90, 91]. Elevated MD and RD are frequently linked to inflammation and demyelination [91**–**93]. The insights gained from these additional analyses are consistent with the main findings of this study and further support the association between MDD, neuroinflammation, and resultant WM structural abnormalities.

Regarding clinical parameters, illness duration was positively correlated with mean ODI values of WM and negatively correlated with mean FA values of WM. Although we lacked data on periods of remission in the MDD group and were therefore unable to account for its potential effects, these findings remain noteworthy. Previous studies have reported negative correlations between illness duration and FA values in regions such as the corpus callosum [94], right superior longitudinal fasciculus, and anterior thalamic radiation [95], as well as reductions in overall mean FA values [40]. These prior studies, however, often focused on specific regions or involved smaller sample sizes. Our study confirmed results consistent with previous research but expanded these findings to the whole-brain level with a larger sample size.

Animal studies also provide relevant insights. Longitudinal imaging studies in rats have shown that control rats exhibit increased FA values with growth, while MDD model rats do not [96]. A longer illness duration suggests that the cells comprising the central nervous system have been exposed to chronic stress for an extended period. This notion is supported by studies in patients with treatment-resistant MDD who have undergone long-term treatment, which have observed elevated microglial activity [97]. The correlations observed in this study between illness duration and diffusion indices (positive for ODI and negative for FA) may reflect the accumulation of WM damage over time, potentially driven by chronic neuroinflammation.

### Relationship between gray and white matter alterations

A visual comparison of the voxelwise analysis results suggested that both GM FWF increases and WM FA reductions may be spatially proximal in regions such as the limbic system and the right temporal lobe to the insular cortex. The regions spanning the limbic system, temporal lobe, and insular cortex, as well as the WM tracts connecting them, are integral components of various neural networks implicated in the pathophysiology of MDD [77, 98]. The present findings therefore suggest that MDD is associated with abnormalities in both the core GM regions constituting the neurocircuitry and the connective WM tissues linking these regions.

As noted, this study did not account for periods of remission when assessing illness duration in patients with MDD. Additionally, being a cross-sectional study, it does not consider the impact of treatment history or the specifics of treatments administered. Future research should focus on drug-naïve patients with first-episode MDD and conduct longitudinal studies to understand better the effects of illness progression and treatment on WM microstructure.

This study is the first to apply NODDI to multi-center data on patients with MDD, enabling a comprehensive analysis of both GM and WM microstructural changes. Our findings provide in vivo evidence suggesting that microstructural tissue changes contribute to underlying pathological processes in the pathophysiology of MDD. This work contributes to understanding the biological underpinnings of the disorder and suggests potential mechanisms that may relate to its clinical presentation. Further investigation into these specific pathological processes could inform the future development of more targeted therapeutic approaches. Continued translational research bridging advanced neuroimaging with cellular and molecular neuroscience is warranted. Such efforts aim to support the refinement of diagnostic approaches and the development of novel treatments, potentially contributing to improved long-term outcomes for patients in clinical settings.

## Supplementary information

Supplementary Materials

## Data Availability

The data that support the findings of this study are available from the corresponding author, upon reasonable request.
