# Supplementary material for: Gray and White matter microstructural alterations in major depressive disorder: a multi-center diffusion imaging study
Source: Transl Psychiatry. 2026 Feb 19;16:163. doi: 10.1038/s41398-026-03916-8 (PMC13022079; doi:10.1038/s41398-026-03916-8)
Supplement: Supplementary file 1 — Supplementary Materials [file 41398_2026_3916_MOESM1_ESM.docx]

**Supplementary Materials**

Supplementary Methods

B0 intensity normalization of diffusion-weighted images.

Supplementary Figures

Supplementary Figure 1. Schematic diagram of diffusion tensor imaging (DTI) and neurite orientation dispersion and density imaging (NODDI) models.

Supplementary Figure 2. Examples of diffusion-derived index maps.

Supplementary Figure 3. Pipeline of tract-based spatial statistics (TBSS) and gray matter-based spatial statistics (GBSS).

Supplementary Figure 4. (A) Regression lines for each diffusion index (mean FWF of GM, mean ODI, and mean FA of WM) with age on the X-axis and the diffusion indices on the Y-axis for both healthy control and depression groups. (B) Interaction plots depicting the interaction between diagnosis (healthy control vs. depression) and sex.

Supplementary Figure 5. Tract-based spatial statistics (TBSS) for group comparisons of MD and RD between healthy controls and patients with MDD.

Supplementary Tables

Supplementary Table 1. MNI coordinates and volumes of clusters (pFWE<0.05; ≥100 mm3) from gray matter-based spatial statistics demonstrating significantly higher gray matter free water fraction (FWF) in individuals with major depressive disorder relative to healthy participants.

Supplementary Table 2. MNI coordinates and volumes of clusters (pFWE<0.05; ≥100 mm3) from tract-based spatial statistics demonstrating significantly lower white matter fractional anisotropy (FA) in individuals with major depressive disorder relative to healthy participants.

Supplementary Table 3. MNI coordinates and volumes of clusters (pFWE<0.05; ≥100 mm3) from tract-based spatial statistics demonstrating significantly higher white matter mean diffusivity (MD) in individuals with major depressive disorder relative to healthy controls.

Supplementary Table 4. MNI coordinates and volumes of clusters (pFWE<0.05; ≥100 mm3) from tract-based spatial statistics demonstrating significantly higher white matter radial diffusivity (RD) in individuals with major depressive disorder relative to healthy controls.

B0 intensity normalization of diffusion-weighted images

The diffusion-weighted imaging (DWI) data processing included a crucial series-wise global intensity normalization step, which is implemented as a standard component of the Human Connectome Project (HCP) pipeline [1]. This procedure ensures that all acquired DWI series (specifically the opposing phase-encoding acquisitions, e.g., anterior-posterior and posterior-anterior) share a consistent overall signal magnitude. This intensity matching is vital for the accuracy of subsequent susceptibility distortion correction using FSL’s “topup” tool.

The normalization was performed as follows:

**1. Reference intensity determination:** The mean signal intensity of the b0 volumes within the first acquired DWI series was calculated to establish the reference mean intensity ($I_{ref}$).

**2. Series Scaling Factor Calculation:** For every subsequent DWI series, a single global scaling factor (SF) was determined by the ratio of the reference intensity to the series intensity ($I_{ref} / I_{series}$).

**3. Application of Global Scaling Factor:** This calculated SF was applied uniformly and identically to every single volume within that series, including all b0 volumes and all diffusion-weighted volumes.

This normalization technique addresses global signal fluctuations (e.g., those caused by scanner drift or minor variations in radiofrequency coil gain between acquisitions) without compromising the biological meaning of the diffusion metrics.

This series-wise global intensity normalization, a standard step in the HCP pipeline, addresses global signal fluctuations (e.g., scanner drift) without compromising the biological meaning of the diffusion metrics. Since the same SF is applied uniformly to all volumes (both $S_{0}$ and $S(b)$), the relative signal ratio $S(b)/S_{0}$, which is the basis for all standard diffusion models (DTI, NODDI, etc.), is preserved. Consequently, the derived quantitative parameters (FA, NDI, ODI) are not systematically biased. Furthermore, this intensity consistency between the opposing phase-encoded b0 images is crucial for the robust performance of the "topup" tool in correcting susceptibility-induced geometric distortions, leading to a more accurate estimation of the brain’s true geometry.

The technical implementation details for the b0 intensity normalization procedure, as well as the entire DWI preprocessing pipeline, are publicly available and can be reviewed in the source code of the Human Connectome Project (HCP) pipelines repository:

Source: <https://github.com/Washington-University/HCPpipelines>

References

1. Glasser MF, Sotiropoulos SN, Wilson JA, Coalson TS, Fischl B, Andersson JL, et al. The minimal preprocessing pipelines for the Human Connectome Project. *Neuroimage.* 2013;80:105–124.

Supplementary Figure 1. Schematic diagram of diffusion tensor imaging (DTI) and neurite orientation dispersion and density imaging (NODDI) models. DTI is a single-compartment model that approximates each voxel as an ellipsoid, assuming a Gaussian distribution of water diffusion. In contrast, NODDI models each voxel as comprising three compartments: intra-neurite (axons and dendrites), extra-neurite (surrounding glial cells and membranes), and free water (cerebrospinal fluid and edema). The neurite density index (NDI) represents the proportion of intra-neurite components within the total tissue. The orientation dispersion index (ODI) measures the angular variation of neurites, indicating how aligned or dispersed the neurite orientations are. The free water fraction (FWF) calculates the proportion of free water components within the total signal, which may indicate regions of edema or cerebrospinal fluid.


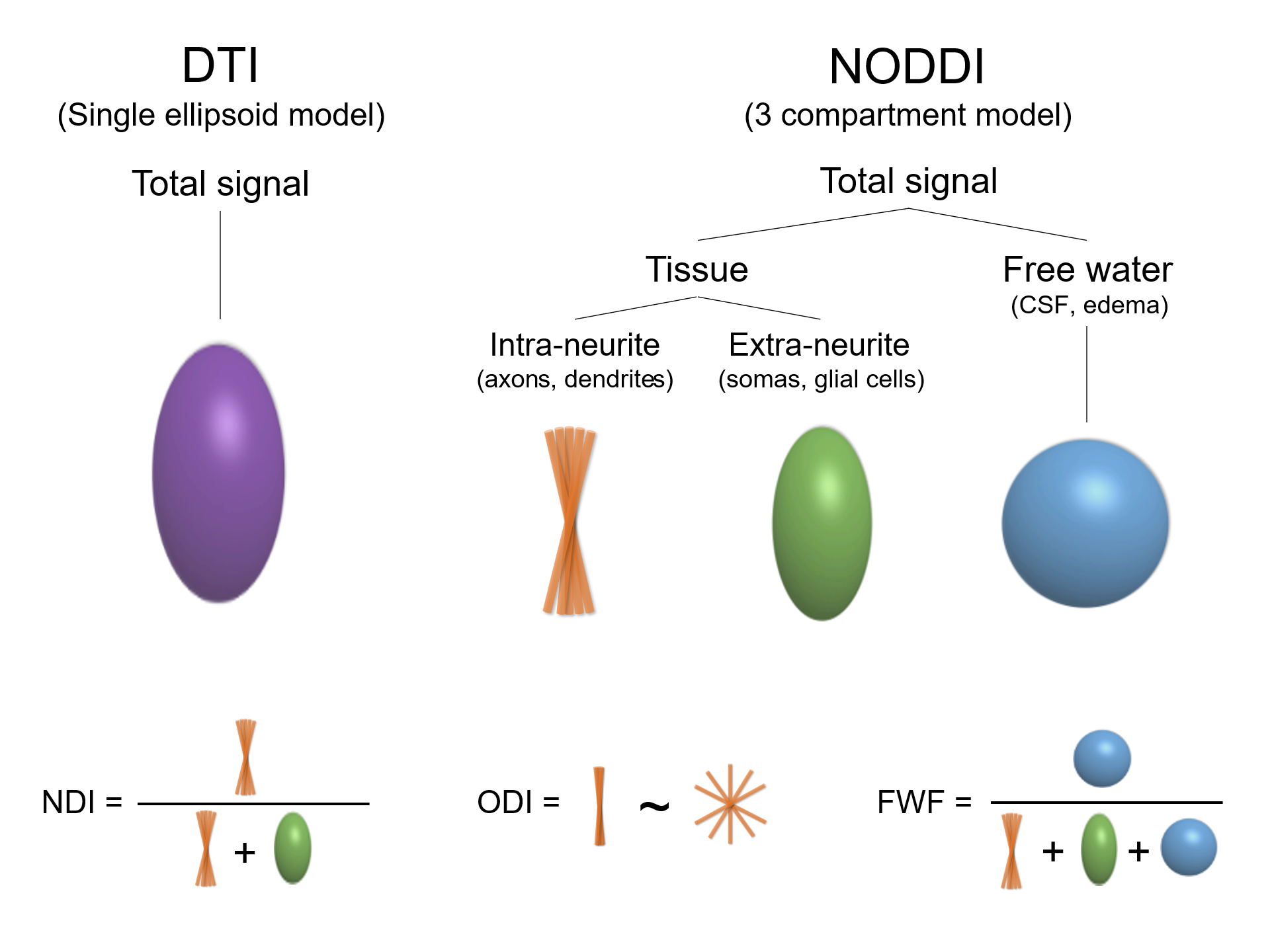


Supplementary Figure 2. Examples of diffusion-derived index maps. The representative axial slices showcase the calculated index maps from a single subject. The maps include Neurite Density Index (NDI), Orientation Dispersion Index (ODI), Free Water Fraction (FWF), Fractional Anisotropy (FA), Mean Diffusivity (MD), Axial Diffusivity (AD), and Radial Diffusivity (RD).


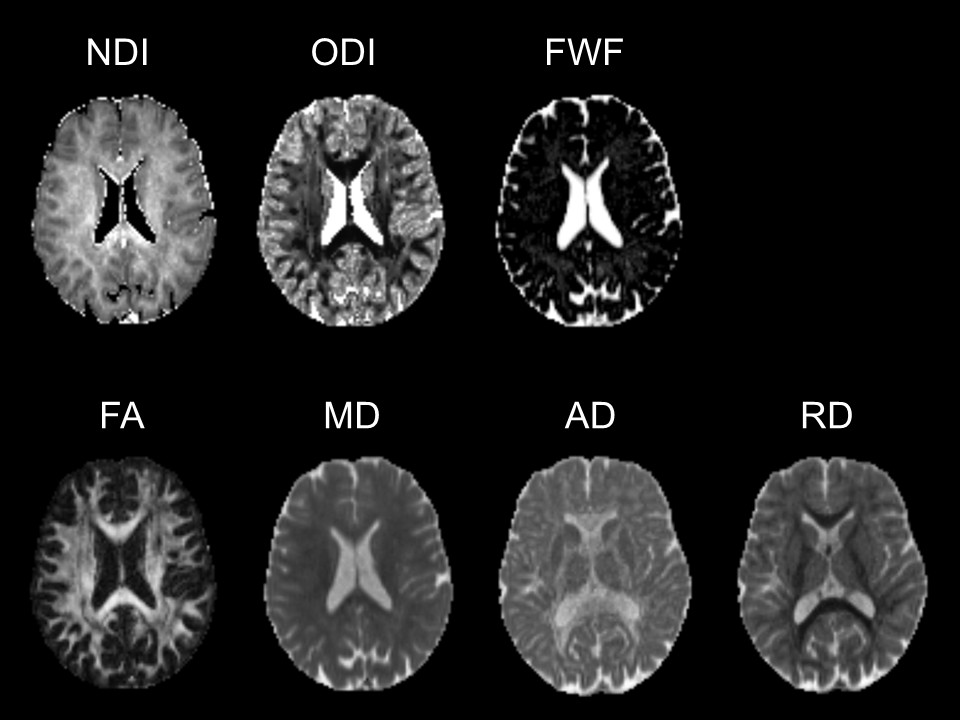


Supplementary Figure 3. Pipeline of tract-based spatial statistics (TBSS) and gray matter-based spatial statistics (GBSS). (A) In TBSS, fractional anisotropy (FA) images of all subjects are registered to the Montreal Neurological Institute (MNI) space to create a mean FA image. This mean FA image is used to generate an FA skeleton mask image, onto which FA values of each subject are projected. Neurite density index (NDI), orientation dispersion index (ODI), and free water fraction (FWF) images are also projected onto the FA skeleton using the same projection vectors. (B) In GBSS, T1-weighted images of all subjects are segmented into gray matter and registered to MNI space to create a mean gray matter image. This mean gray matter image is used to generate a gray matter skeleton mask image, onto which NDI, ODI, and FWF images are projected for gray matter analysis.


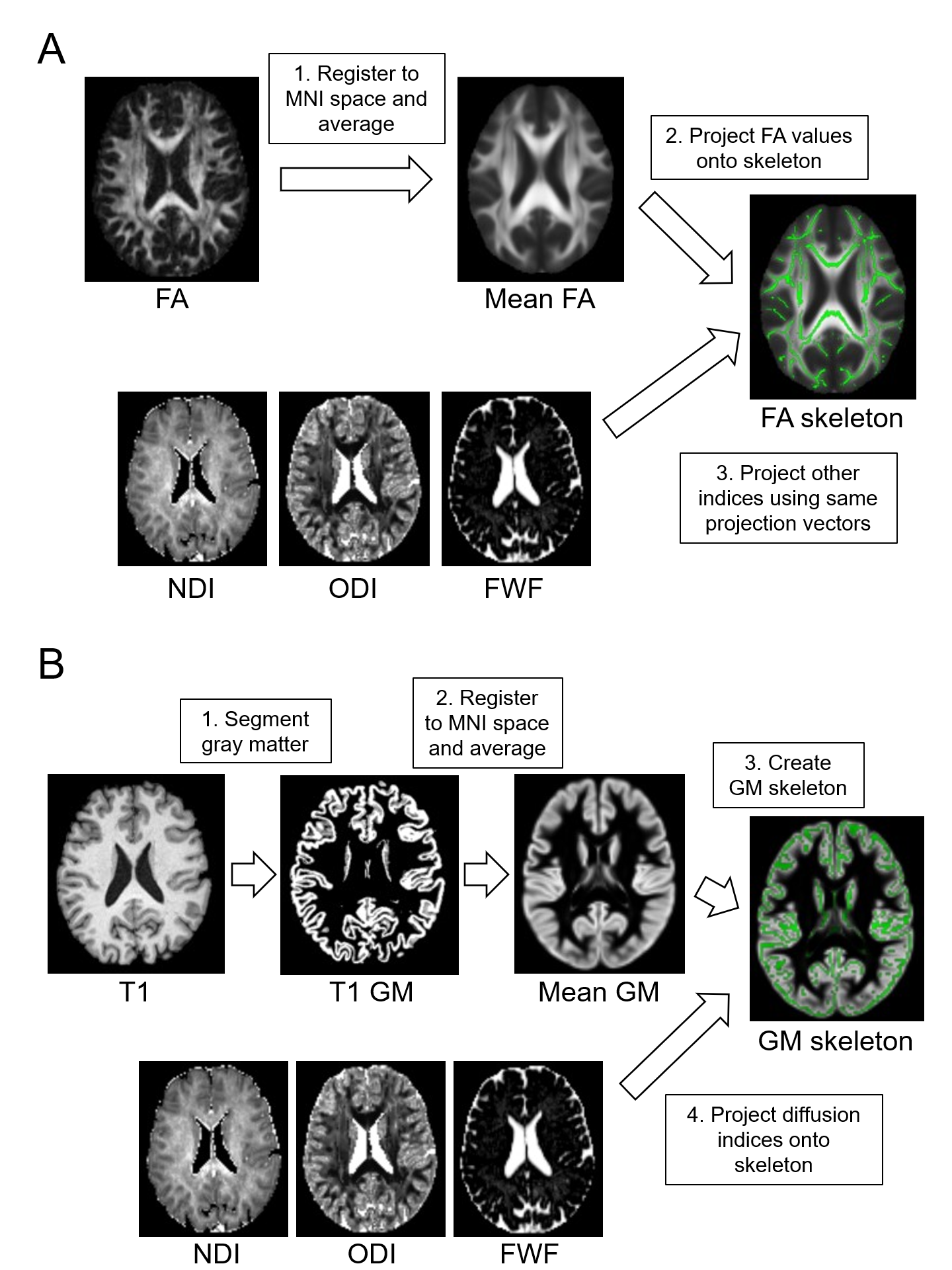


Supplementary Figure 4. (A) Regression lines for each diffusion index (mean FWF of GM, mean ODI and mean FA of WM) plotted against age. The X-axis represents age, and the Y-axis represents the diffusion indices for both healthy control and depression groups. (B) Interaction plots showing the interaction between diagnosis (healthy control vs. depression) and sex on diffusion indices.


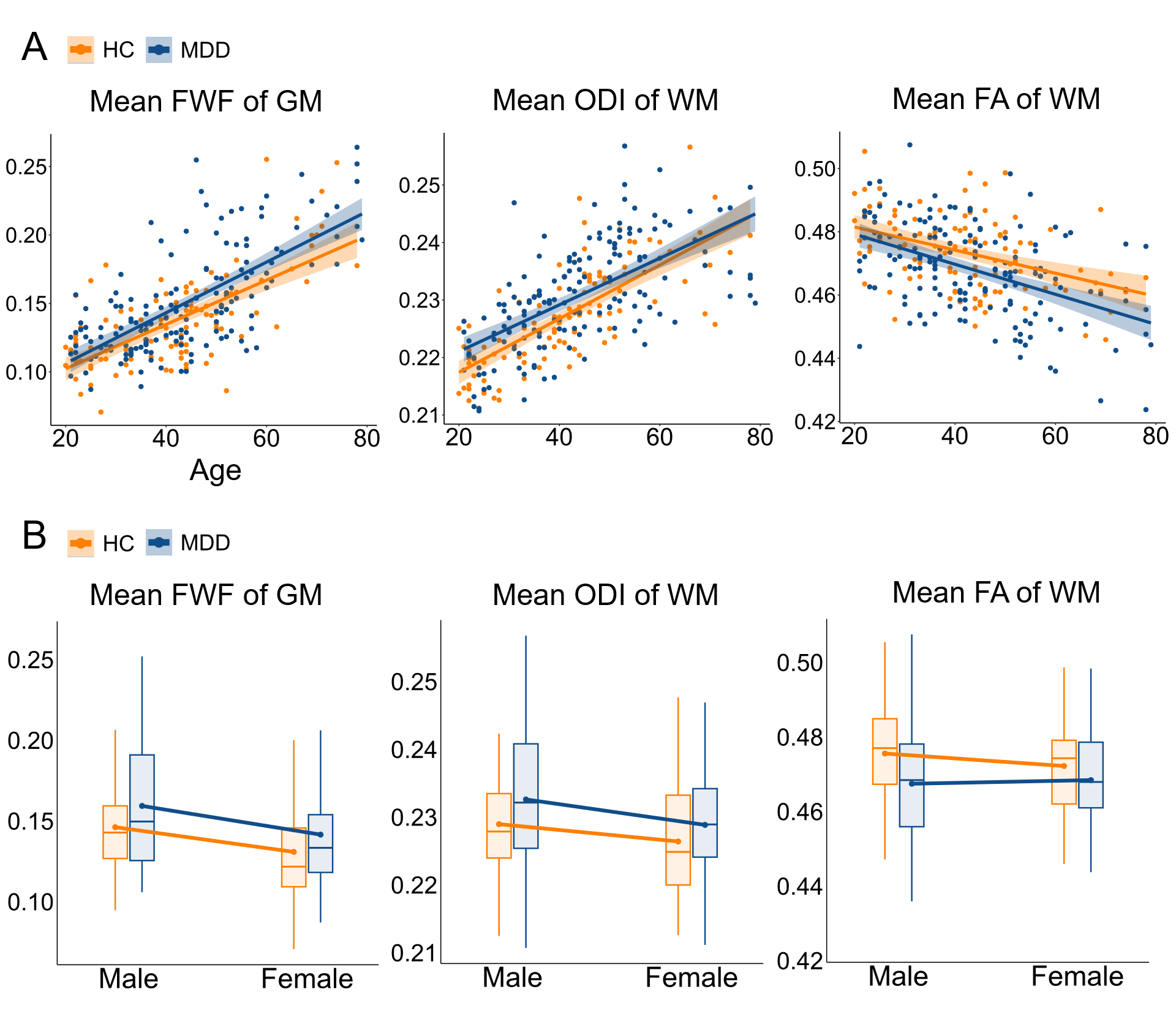


Supplementary Figure 5. Tract-based spatial statistics (TBSS) for group comparisons of MD and RD between the healthy controls and the patients with MDD. TBSS revealed increases in white matter MD (red-yellow voxels), and in white matter RD. The “tbss_fill” command in FSL was used to enhance visibility of the significant tracts.
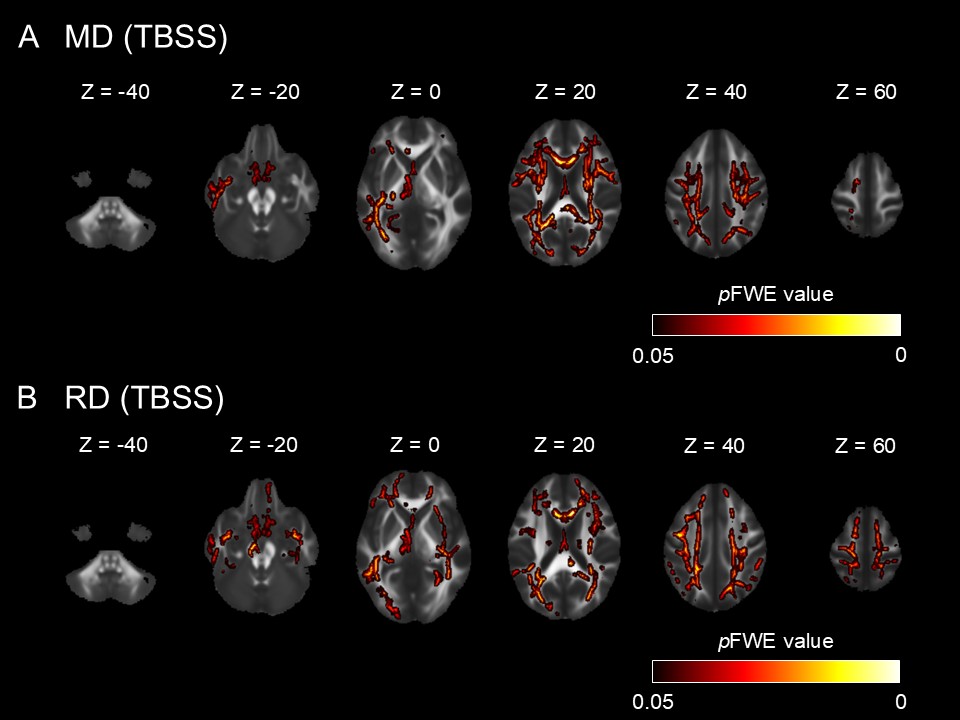


Supplementary Table 1. MNI coordinates and volumes of clusters (pFWE<0.05; ≥100 mm3) from gray matter-based spatial statistics showing significantly higher gray matter free water fraction (FWF) in individuals with major depressive disorder relative to healthy participants.

|  |  |  | MNI coordinates (mm) | | |  |
| --- | --- | --- | --- | --- | --- | --- |
| Cluster | Volume(mm^3^) | Minimum *p* value | X | Y | Z | Brain regions |
| FWF1 | 24,912 | 0.008 | 20 | -46 | 0 | R TOF, L TOF, R PrG, R PhGa, L PhGa, R PhGp, L PhGp, R MTGa,  R LG, L LG, R TP, L TP, R STGa,  R PcC, R TFCp, L TFCp, R ScC,  R TFCa, L TFCa, R FP, R MTGt,  R FOC, R PT, R Ins, R ITGp, L ITGp,  R POpC, R MTGp, R OFC, R OFG,  L OFG, R COpC, R IFGpt, R CGp,  R ITGa, R H1/H2, R PP, R HIP, L HIP,  R AMG, L AMG, R CAU, L CAU,  R THA, L THA, R BS, L BS, R NAc,  L NAc, |
| FWF2 | 1,032 | 0.039 | -6 | -32 | 26 | L CGp, L PrG, L PcC |
| FWF3 | 392 | 0.04 | -36 | -2 | 14 | L COpC, L POpC, L Ins |
| FWF4 | 264 | 0.042 | 68 | -30 | 12 | R STGp |
| FWF5 | 168 | 0.044 | -62 | -20 | -22 | L MTGp, L MTGa |
| FWF6 | 136 | 0.043 | -66 | -36 | -10 | L ITGp, L MTGp |
| FWF7 | 136 | 0.049 | -14 | -60 | 8 | L PcC, L SccC |
| FWF8 | 112 | 0.045 | -36 | -16 | 4 | L Ins |
| R, right; L, left; TOF, temporal occipital fusiform cortex; PrG, precentral gyrus; PhGa, parahippocampal gyrus, anterior division; PhGp, parahippocampal gyrus, posterior division; MTGa, middle temporal gyrus, anterior division; LG, lingual gyrus; TP, temporal pole; STGa, superior temporal gyrus, anterior division; PcC, precuneous cortex; TFCp, temporal fusiform cortex, posterior division; ScC, subcallosal cortex; TFCa, temporal fusiform cortex, anterior division; FP frontal pole; MTGt, middle temporal gyrus, temporooccipital part; FOC frontal opercular cortex; PT, planum temporale; Ins, insular cortex; ITGp, inferior temporal gyrus, posterior division; POpC, parietal opercular cortex; MTGp, middle temporal gyrus, posterior division; OFC, orbitofrontal cortex; OFG, occipital fusiform gyrus; COpC, central opercular cortex; IFGpt, inferior frontal gyrus, pars triangularis; CGp, cingulate gyrus, posterior division; ITGa, inferior temporal gyrus, anterior division; H1/H2, Heschl’s gyrus (includes H1 and H2); PP, planum polare; STGp, superior temporal gyrus, posterior division; SccC, supracalcarine cortex; HIP, hippocampus; AMG, amygdala; THA, thalamus; CAU, caudate; BS, brain stem; NAc, nucleus accumbens. | | | | | | |

Supplementary Table 2. MNI coordinates and volumes of clusters (pFWE<0.05; ≥100 mm3) from tract-based spatial statistics showing significantly lower white matter fractional anisotropy (FA) in individuals with major depressive disorder relative to healthy participants.

|  |  |  | MNI coordinates (mm) | | |  |
| --- | --- | --- | --- | --- | --- | --- |
| Cluster | Volume(mm^3^) | Minimum *p* value | X | Y | Z | Brain regions |
| FA1 | 38,675 | 0.003 | 11 | -19 | 28 | R CST, L CST, R PCR, L PCR, L FX/ST,  R FX/ST, R CGC,  L CGC, MCP, R SLF, L SLF,  L TAP, R SCR, L SCR, R EC,  L EC, R SCP, L SCP, R CGH,  L CGH, R PLIC, R RLIC, L RLIC, R ALIC, L ALIC, L PLIC, SCC,  R SS, L SS, R PTR, L PTR, L CP, R UNC, R SFO, L SFO, FX,  R IFO, L IFO, L ACR |
| R, right; L, left; CST, corticospinal tract; PCR, posterior corona radiata; FX/ST, fornix (cres) / stria terminalis; CGC, cingulum (cingulate gyrus); MCP, middle cerebellar peduncle; SLF, superior longitudinal fasciculus; TAP, tapetum; SCR, superior corona radiata; EC, external capsule; SCP, superior cerebellar peduncle; CGH, cingulum (hippocampus); RLIC, retrolenticular part of internal capsule; ALIC, anterior limb of internal capsule; PLIC, posterior limb of internal capsule; SCC, splenium of corpus callosum; SS, sagittal stratum; PTR, posterior thalamic radiation; CP, cerebral peduncle; UNC, uncinate fasciculus; SFO, superior fronto-occipital fasciculus; FX, fornix (column and body of fornix); IFO, inferior fronto-occipital fasciculus; ACR, anterior corona radiata. | | | | | | |

Supplementary Table 3. MNI coordinates and volumes of clusters (pFWE<0.05; ≥100 mm3) from tract-based spatial statistics demonstrating significantly higher white matter mean diffusivity (MD) in individuals with major depressive disorder relative to healthy participants.

|  |  |  | MNI coordinates (mm) | | |  |
| --- | --- | --- | --- | --- | --- | --- |
| Cluster | Volume(mm^3^) | Minimum *p* value | X | Y | Z | Brain regions |
| MD1 | 29,280 | 0.009 | 9 | 11 | 24 | R PTR, L PTR, R EC, L EC, R PCR,  L PCR, R TAP, L TAP, R CGH, FX,  R PLIC, L PLIC, R RLIC, L RLIC, R IFO,  R CGC, L CGC, R ACR, L ACR, R UNC,  L FX/ST, R SFO, L SFO, BCC, R CST,  R SCR, L SCR, R SS, MCP, L ALIC,  L SLF, SCC |
| MD2 | 2,736 | 0.042 | 1 | 4 | -25 | SCC, R SCP, L SCP, R ICP, L ICP,  R FX/ST, R ML, L ML, L CGH, MCP,  L ALIC, R CST, R RLIC, FX, R PLIC,  L PLIC, R CP |
| R, right; L, left; PTR, posterior thalamic radiation; EC, external capsule; PCR, posterior corona radiata; TAP, tapetum; CGH, cingulum (hippocampus); FX, fornix (column and body of fornix); PLIC, posterior limb of internal capsule; RLIC, retrolenticular part of internal capsule; IFO, inferior fronto-occipital fasciculus; CGC, cingulum (cingulate gyrus); ACR, anterior corona radiata; UNC, uncinate fasciculus; FX/ST, fornix (cres) / stria terminalis; SFO, superior fronto-occipital fasciculus; BCC, body of corpus callosum; CST, corticospinal tract; SCR, superior corona radiata; SS, sagittal stratum; MCP, middle cerebellar peduncle; ALIC, anterior limb of internal capsule; SLF, superior longitudinal fasciculus; SCC, splenium of corpus callosum; SCP, superior cerebellar peduncle; ICP, inferior cerebellar peduncle; ML, medial lemniscus; PLIC, posterior limb of internal capsule; CP, cerebral peduncle. | | | | | | |

Supplementary Table 4. MNI coordinates and volumes of clusters (pFWE<0.05; ≥100 mm3) from tract-based spatial statistics demonstrating significantly higher white matter radial diffusivity (RD) in individuals with major depressive disorder relative to healthy participants.

|  |  |  | MNI coordinates (mm) | | |  |
| --- | --- | --- | --- | --- | --- | --- |
| Cluster | Volume(mm^3^) | Minimum *p* value | X | Y | Z | Brain regions |
| RD1 | 36,309 | 0.004 | 14 | -17 | 31 | R PTR, L PTR, R SCP, L SCP, R FX/ST,  L FX/ST, R EC, L EC, R ML, L ML,  R PCR, L PCR, R TAP, L TAP, R UNC,  L UNC, R CGH, L CGH, FX, R PLIC,  L PLIC, R RLIC, L RLIC, R IFO, L IFO,  R CGC, L CGC, PCT, R ICP, L ICP,  R ALIC, L ALIC, GCC, R SFO, L SFO,  R SS, L SS, BCC, R CST, R SCR, L SCR,  L ACR, MCP, R SLF, L SLF, SCC, R CP |
| R, right; L, left; PTR, posterior thalamic radiation; SCP, superior cerebellar peduncle; FX/ST, fornix (cres) / stria terminalis; EC, external capsule; ML, medial lemniscus; PCR, posterior corona radiata; TAP, tapetum; UNC, uncinate fasciculus; CGH, cingulum (hippocampus); FX, fornix (column and body of fornix); PLIC, posterior limb of internal capsule; RLIC, retrolenticular part of internal capsule; IFO, inferior fronto-occipital fasciculus; CGC, cingulum (cingulate gyrus); PCT, pontine crossing tract; ICP, inferior cerebellar peduncle; ALIC, anterior limb of internal capsule; GCC, genu of corpus callosum; SFO, superior fronto-occipital fasciculus; SS, sagittal stratum; BCC, body of corpus callosum, CST, corticospinal tract; SCR, superior corona radiata; ACR, anterior corona radiata; MCP, middle cerebellar peduncle; SLF, superior longitudinal fasciculus; SCC, splenium of corpus callosum; CP, cerebral peduncle. | | | | | | |
